# Association of moderate and vigorous physical activity and relative muscle strength with neck circumference: a cross-sectional analysis of the Study of Health in Pomerode (SHIP-Brazil)

**DOI:** 10.31744/einstein_journal/2023AO0186

**Published:** 2023-05-23

**Authors:** Clóvis Arlindo de Sousa, Marcello Ricardo Paulista Markus, Karina Passero, Laís Carolini Theis, Alan de Jesus Pires de Moraes, Quelen Schutz Carvalho Bernardes Malafaia, Ernani Tiaraju de Santa Helena

**Affiliations:** 1 Universidade Regional de Blumenau Blumenau SC Brazil Universidade Regional de Blumenau, Blumenau, SC, Brazil.; 2 University Medicine Greifswald Greifswald Germany University Medicine Greifswald, Greifswald, Germany.; 3 Universidade do Vale do Itajaí Itajaí SC Brazil Universidade do Vale do Itajaí, Itajaí, SC, Brazil.

**Keywords:** Neck, Exercise, Sedentary behavior, Hand strength, Measurement, Anthropometry, Epidemiology, Risk factors

## Abstract

**Objective:**

Neck circumference is a simple anthropometric measurement that may be linked to chronic diseases, physical activity, and muscle strength. We sought to verify the association of moderate and vigorous physical activity levels and relative muscle strength with neck circumference in a community in southern Brazil.

**Methods:**

We cross-sectionally analyzed data from 2,488 participants (51% women), aged 20-79 years old from the Study of Health in Pomerode (SHIP-Brazil) conducted in Pomerode, Santa Catarina, Brazil. Increased neck circumference was defined with cutoff points of >39cm for men and >35cm for women. The independent variables were the level of moderate and vigorous physical activity using the short International Physical Activity Questionnaire, and relative muscle strength using the handgrip test and body mass. Univariate and multiple Poisson regression models were used to determine the association between variables (p≤0.05).

**Results:**

The prevalence of increased neck circumference was 48.2% (60.4% in men, 39.6% in women) and was associated with low relative muscle strength (PR=1.26, 95%CI: 1.17-1.35) in men, insufficient moderate and vigorous physical activity levels (PR=1.23, 95%CI: 1.14-1.32), and relative muscle strength (PR=1.73, 95%CI: 1.61-1.87) in women. After adjusting for covariates, no significant associations were observed between insufficient moderate and vigorous physical activity levels in men (PR=1.02, 95%CI: 0.95-1.07).

**Conclusion:**

Increased neck circumference seems to be an important predictor of low moderate and vigorous physical activity and relative strength loss in adults, and more pronounced in women.

**Figure:**
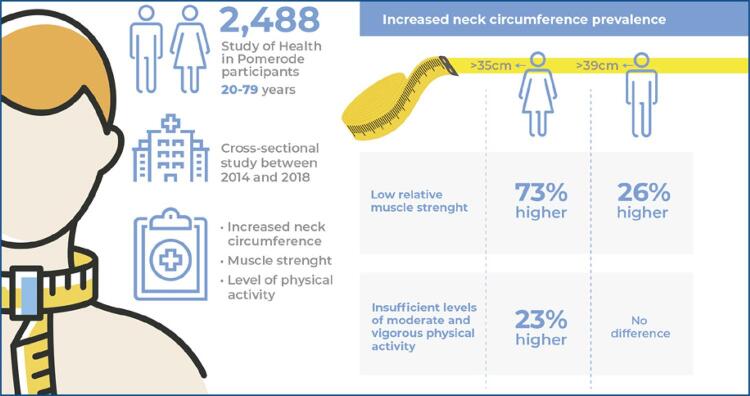

## INTRODUCTION

Physical inactivity and obesity are major global public health concerns,^( 1 , 2 )^ and are both determinants of premature mortality.^( 3 )^ Adipose tissue accumulation in the upper body is strongly associated with cardiometabolic diseases.^( 4 )^ Additionally, greater central obesity, regardless of physical activity, is related to a higher chance of low relative muscle strength (RMS) since adolescence.^( 5 )^

The neck circumference (NC) is a simple anthropometric measurement associated with levels of physical activity, and can be measured more easily than waist circumference.^( 6 , 7 )^ Moreover, NC showed similar or better association with metabolic factors and may be used in special populations such as morbidly obese people, patients in bed rest, ostomates, and pregnant women.^( 6 )^ Neck circumference is the most appropriate anthropometric marker for identifying fat distribution associated with high cardiometabolic risk.^( 8 )^ However, despite its potential as a good anthropometric indicator for different diseases, health outcomes, and lifestyle, NC has been rarely studied, and current evidence remains incomplete.^( 9 )^

In this context, the decrease in moderate and vigorous physical activity (MVPA) with age^( 10 )^ may be associated with increased weakness^( 11 )^ and body adiposity,^( 12 , 13 )^ and decreased muscle strength.^( 11 )^ People with dynapenic-abdominal obesity are weak and more likely to fall.^( 13 )^ This vicious cycle can be stopped by a sufficient level of MVPA.^( 11 , 14 , 15 )^

Few studies have shown an association between NC and RMS,^( 16 )^ sedentary behavior^( 15 )^ and MVPA in selected countries.^( 7 )^ However, to our knowledge, no study has verified the association of NC with MVPA and RMS in older adult population, stratified according to sex.

## OBJECTIVE

We investigated the association of neck circumference with moderate and vigorous physical activity and relative muscle strength in older adults from a community in Southern Brazil.

## METHODS

### Study population

We cross-sectionally analyzed the baseline data of the Study of Health in Pomerode, SHIP-Brazil, conducted between 2014 and 2018. Pomerode is a city with 34,000 inhabitants in the state of Santa Catarina, in Southern Brazil. It was founded in the 19^th^ century by the Pomeranian immigrants.^( 17 )^ The SHIP-Brazil is a sister study to the Study of Health in Pomerania (SHIP) conducted in Germany.^( 18 , 19 )^ For SHIP-Brazil, we performed the translation, preparation of training manuals, standard operating procedures (SOPs), and questionnaires from SHIP. Training with this material was maintained throughout data collection. All SHIP-Brazil interviewers and examiners were trained and certified according to SHIP standards.

Participants were identified from simple random sampling across 12 strata of both sexes, aged 20-79 years, with 10-year intervals. The sample calculation considered a prevalence of events of 50%, precision of 5%, and 95% confidence interval (95%CI). The sample was drawn from 3,678 people residing in Pomerode, Santa Catarina, Brazil for at least six months. Individuals with physical or mental limitation that prevented them from answering the questionnaires or carrying out health examinations, and those that refuse to sign the written informed consent form, were excluded. Additionally, in the functional measures sector, those who had any limitations or difficulties in carrying out measures, such as pregnancy, wheelchair users, use of ostomy bags, wounds, or hernias at measurement sites, were excluded. Approximately 30% of losses and refusals occurred. The total sample consisted of 2,488 participants.

All participants were informed about the purpose and procedures, and signed a written consent form after agreeing. The study was conducted in accordance with the Declaration of Helsinki for medical research involving humans and approved by the Ethics Committee of *Universidade de Blumenau* , Blumenau, SC, Brazil (CAAE: 24998019.4.0000.5370; # 3.718.309).

### Interview and examinations

#### Dependent variable

Neck circumference was measured with the participant standing, head positioned in the horizontal plane of the Frankfurt. It was measured at the midpoint of the neck, just below the thyroid cartilage, using an inelastic tape.^( 20 )^ High cut-off points of >39cm for men and >35cm for women^( 7 )^ were used.

## Independent variables

### Relative muscle strength

We estimated the absolute muscle strength by handgrip strength (HGS), and was measured using a handgrip dynamometer (Jamar Plus Digital Dynamometer, Patterson Medical, Sammons Preston, Bolingbrook, IL). The test was performed with the participants sitting on a chair without touching their backs against the backrest, feet flat on the floor, or on a bench in the case of short stature. The shoulders and forearms were in a neutral position with the elbows in 90^o^ flexion and with calm breathing without holding. Upon command, three measures were obtained for each hand, with six measures overall. Three measurements were taken with the right hand interspersed with the left. The time interval between measurements was at least 15 seconds. All six readings were recorded, and the highest value obtained during the measurements was used for the present study.^( 21 , 22 )^ To obtain RMS, the following formula was used (RMS = absolute strength (kg) / body mass (kg)).^( 16 )^ Relative muscle strength adjusted to body size can provide more accurate information for screening sarcopenic obesity.^( 23 )^ The lower quartile was used to obtain the cut-off point for this variable, with the cut-off point of the RMS being low at ≤0.42 for men and at ≤0.28 for women.

## Moderate and vigorous physical activity

To estimate the level of MVPA, we used the short version of the International Physical Activity Questionnaire.^( 24 )^ The weekly minutes of moderate physical activity (PA) were added to twice the minutes of vigorous PA. Participants who perform MVPA for 150 minutes a week or less were categorized as sufficiently or insufficiently active, respectively.^( 25 )^

## Adjustment variables

The adjustment variables were obtained from a face-to-face questionnaire and exams, and consist of sex (female, male), age group (20-29, 30-39, 40-49, 50-59, 60-69 or 70-79 years), school education (0, 1-8, 9-11 or ≥12 years), German culture (yes or no; participants speak the German language at home and participate in German socio-cultural associations), smoking status (never, former, or current smoker), alcohol consumption (low, moderate, high, or severe), waist/hip ratio (no <0.90cm man and <0.85cm women, or yes ≥0.90cm man and ≥0.85cm women), and multimorbidity (0-1 or 2+ chronic diseases (hypertension, myocardial infarction, stroke, diabetes, and dyslipidemia).

## Statistical analysis

Descriptive data were estimated using prevalence and 95%CI for the whole sample and stratified by NC and sex. Age and NC are also described as mean ± standard deviation and median [25-75% interquartile range]. The associations between NC and other variables were based on the χ^2^ test. The analyses of the associations between the independent variables (RMS and MVPA) and the outcomes were based on the prevalence ratio (PR) and 95%CI estimated using univariate and multiple Poisson regression adjusted for model 1 (adjusted for RMS and MVPA levels); model 2 (adjusted for model 1 and age group, school education, and German culture); and model 3 (adjusted for model 1, model 2, smoking status, alcohol consumption, waist/hip ratio, and multimorbidity). All statistical analysis were performed using SPSS version 22.0 (IBM Corp., Armonk, NY, USA). Differences were considered to be statistically significant at p≤0.05.

## RESULTS

**Figure 1:**
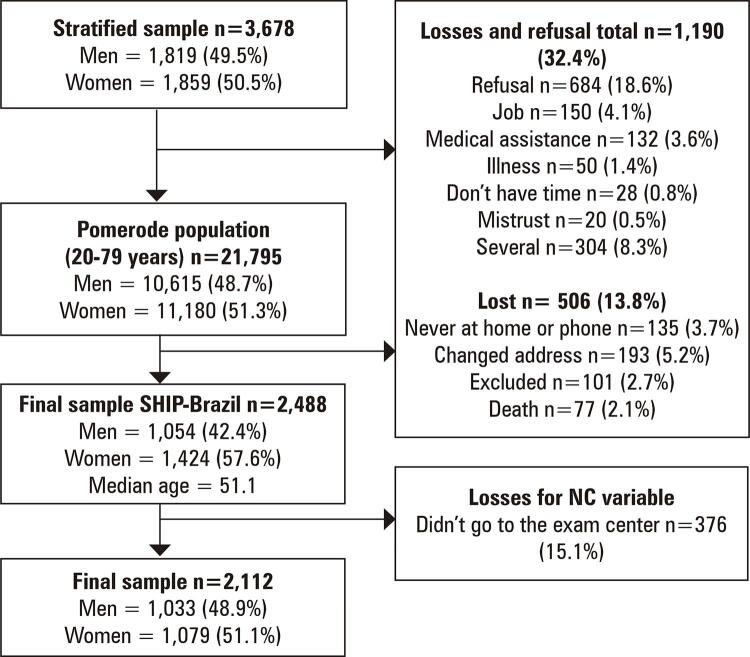
Participants flow chart NC: neck circumference.

We included data of 2,488 individuals aged 20-79 years. There were losses and refusals for NC (n=376, 15.1%), MVPA levels (n=335, 13.5%), and RMS (n=410, 16.5%). Losses and refusals were more prevalent in those without education, current smokers, with multimorbidity, and with low RMS (p<0.05). There was no difference in the mean age between participants (50.9±14.6 years) and non-participants (49.4±16.4 years) due to losses (p=0.570). Considering the dependent variable NC, a total of 2,112 individuals aged 20-79 years were included for the final analyses ( Figure 1 ).

The mean age was 43.2±14.43 years for men and 44.1±14.96 years for women. The participants were comprised of 48.9% men and 51.1% women, with school education of 1-8 years (45.6%) and 9-12 years (32.1%). Preserve German culture accounted for 64.5% of the participants. The prevalence of current smokers was 9.8%, and high and severe alcohol consumption was 9.1% and 6.1%, respectively. The population presented with 46.7% waist/hip ratio ≥0.90 for men and ≥0.85 for women, 14.8% with more than two chronic diseases, 26.7% were insufficiently active, and 21.4% RMS ≤0.42 and ≤0.28 were recorded for men and women, respectively.

The prevalence of increased NC was higher in males (60.4%), aged group 50-59 (19%) and 60-69 years (13.5%), school education 1-8 years (52%), presence of German culture (68%), former smoker (21.8%), high (12.1%) and severe (8.6%) alcohol consumption, high waist-to-hip ratio (70.2%), >2 chronic disease (21.2%), insufficiently active MVPA (29.7%), and low RMS (32.4%) ( Table 1 ).

**Table 1:** Prevalence stratified by neck circumference and univariate analysis

Variables	Total sample (n=2,112) %	Neck circumference		p value*

		Normal (n=1,044)	Increased (n=1,068)	
	(95%CI)	(95%CI)	(95%CI)	
Sex (n=2,112)				<0.001
Female	51.1 (50.1-52.1)	61.9 (59.3-64.4)	39.6 (37.1-42.1)	
Male	48.9 (47.9-49.9)	38.1 (35.5-40.7)	60.4 (57.9-62.9)	
Age group (years) (n=2,112)				<0.001
20-29	21.3 (20.2-22.4)	28.2 (25.8-30.8)	13.8 (11.4-16.5)	
30-39	24.1 (23.2-25.0)	24.0 (21.8-26.3)	24.2 (21.9-26.7)	
40-49	21.6 (20.9-22.4)	20.5 (18.7-22.4)	22.9 (20.9-24.9)	
50-59	16.5 (16.0-17.1)	14.2 (12.8-15.7)	19.0 (17.5-20.7)	
60-69	10.6 (10.2-11.1)	8.0 (7.0-9.1)	13.5 (12.3-14.7)	
70-79	5.8 (5.5-6.1)	5.1 (4.3-5.9)	6.7 (5.9-7.6)	
School education (years) (n=2,112)				<0.001
0	0.8 (0.5-1.1)	0.9 (0.5-1.5)	0.7 (0.4-1.2)	
1-8	45.6 (43.5-47.6)	39.5 (36.6-42.5)	52.0 (48.8-55.3)	
9-12	32.1 (29.8-34.4)	35.3 (32.0-38.7)	28.6 (25.4-31.9)	
≥12	21.6 (19.6-23.8)	24.3 (21.4-27.5)	18.7 (15.9-21.8)	
German culture (n=2,078)				<0.001
No	35.5 (33.2-37.9)	38.8 (35.5-42.3)	32.0 (28.7-35.4)	
Yes	64.5 (62.1-66.8)	61.2 (57.7-64.5)	68.0 (64.6-71.3)	
Smoking status (n=2,071)				0.007
Never smoker	71.1 (68.9-73.2)	74.3 (71.3-77.1)	67.6 (64.4-70.7)	
Former smoker	19.1 (17.4-21.0)	16.7 (14.4-19.2)	21.8 (19.2-24.6)	
Current smoker	9.8 (8.4-11.4)	9.0 (7.2-11.1)	10.6 (8.6-13.0)	
Alcohol consumption (n=2,064)				<0.001
Low	65.5 (63.2-67.7)	69.8 (66.5-73.0)	60.8 (57.4-64.1)	
Moderate	19.3 (17.4-21.4)	20.1 (17.4-23.1)	18.5 (15.8-21.4)	
High	9.1 (7.8-10.7)	6.4 (4.8-8.4)	12.1 (9.9-14.8)	
Severe	6.1 (4.9-7.5)	3.7 (2.5-5.6)	8.6 (6.7-11.1)	
Waist/hip ratio (n=2,105)				<0.001
No (<0.90 men; <0.85 women)	53.3 (51.2-55.4)	75.1 (72.5-77.6)	29.8 (26.6-33.1)	
Yes (≥0.90 men; ≥0.85 women)	46.7 (44.6-48.8)	24.9 (22.4-27.5)	70.2 (66.9-73.4)	
Multimorbidity (n=1,908)				<0.001
0-1	85.2 (83.7-86.6)	91.2 (89.5-92.7)	78.8 (76.2-81.2)	
2+ chronic diseases	14.8 (13.4-16.3)	8.8 (7.3-10.5)	21.2 (18.8-23.8)	
MVPA (min./week) (n=2,037)				0.001
Active (≥150)	73.3 (71.1-75.4)	76.1 (73.0-78.9)	70.3 (67.0-73.4)	
Insufficiently active (<150)	26.7 (24.6-28.9)	23.9 (21.1-27.0)	29.7 (26.6-33.0)	
Low RMS (n=2,069)				<0.001
No (>0.42 men; >0.28 women)	78.6 (76.6-80.5)	88.9 (86.6-90.9)	67.6 (64.5-70.6)	
Yes (≤0.42 men; ≤0.28 women)	21.4 (19.5-23.4)	11.1 (9.1-13.4)	32.4 (29.4-35.5)	

* p values are based on the χ^2^ test for categorical variables.

MVPA: moderate and vigorous physical activity; RMS: relative muscle strength; 95%CI: 95% confidence interval.

**Figure 2:**
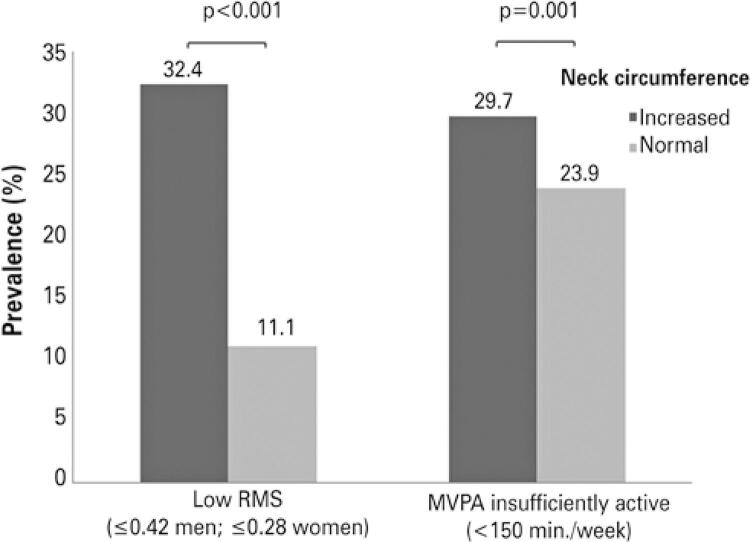
Prevalence of increased neck circumference by low relative muscle strength and insufficient levels of moderate and vigorous physical activity RMS: relative muscle strength; MVPA: moderate and vigorous physical activity.

The mean NC was 40±3.21cm in men and 34.4±2.90cm in women. The median NC was 39.7cm (37.8-42cm) in men and 34.1cm (32.3-36.3cm) in women. Figure 2 shows the differences in the prevalence of increased and normal NC according to low RMS (p<0.001) and insufficient MVPA levels (p=0.001). The low RMS presented a more significant difference for the NC group.

Finally, in multiple regression analyses, we showed the PR and 95%CI of factors independently associated with NC stratified by sex. In women, after adjusting for all variables, low RMS (PR=1.73, p<0.001) and insufficient MVPA levels (PR=1.23, p<0.001) remained associated with NC. Conversely in men, low RMS (PR=1.26, p<0.001) was associated with NC; however, insufficient MVPA levels (PR=1.02, p=0.627) showed no significant association after adjusting for health conditions, demographic, and lifestyle variables ( Table 2 ).

**Table 2:** Poisson regression model for increased neck circumference according to low relative muscle strength and insufficient moderate and vigorous physical activity levels stratified by sex

		Men (n=900)			Women (n=1,212)		
	
		RP	95%CI	p value	RP	95%CI	p value
Not adjusted	Low RMS	1.45	1.37-1.54	<0.001	2.34	2.18-2.51	<0.001
	MVPA insufficiently active	1.12	1.05-1.19	<0.001	1.31	1.22-1.41	<0.001
Model 1	Low RMS	1.43	1.34-1.52	<0.001	2.32	2.16-2.49	<0.001
	MVPA insufficiently active	1.08	1.01-1.15	0.017	1.27	1.18-1.36	<0.001
Model 2	Low RMS	1.52	1.43-1.62	<0.001	2.16	2.01-2.32	<0.001
	MVPA insufficiently active	1.06	0.96-1.13	0.071	1.28	1.19-1.38	<0.001
Model 3	Low RMS	1.26	1.17-1.35	<0.001	1.73	1.61-1.87	<0.001
	MVPA insufficiently active	1.02	0.95-1.07	0.627	1.23	1.14-1.32	<0.001

PR: prevalence ratio; RMS: relative muscle strength; MVPA: moderate and vigorous physical activity; MVPA: insufficiently active; Model 1: adjusted for low RMS and MVPA insufficiently active; Model 2: adjusted for model 1 and age group, school education, and German culture; Model 3: adjusted for model 1 and model 2 and smoking status, alcohol consumption, waist/hip ratio, and multimorbidity.

## DISCUSSION

In this study, we investigated the association of NC with MVPA and RMS among older adults in Southern Brazil. We found that increased NC was high in this population, especially in men. Moreover, our results showed an association between MVPA and RMS and NC in women and between RMS and NC in men.

Neck circumference is a simple and practical anthropometric parameter that can be measured more easily than other parameters, such as waist, abdomen, and hip circumference.^( 6 , 26 )^ It is an appropriate anthropometric marker to identify the distribution of fat associated with features of cardiometabolic risk and chronic diseases in women with severe obesity (n=305; mean age, 43 years).^( 8 )^ Additionally, it has been shown as a best anthropometric measure associated with metabolic risk markers in Hispanics without cardiovascular diseases (n=1,206 participants, 40-65 years old, both sexes).^( 6 )^ Researchers in the Framingham Heart Study cohorts suggested using NC as a new measure of cardiometabolic risk owing to its association with circumference and cardiovascular disease risk factors, even after adjusting for body fat composition.^( 27 )^ The Brazilian Metabolic Syndrome Study (BRAMS) was conducted in several regions in Brazil with 1,053 adult participants (28.6% men, 18-60 years old). The authors concluded that NC measures involve an innovative and alternative approach to estimate body fat and the risk factors associated with the components of metabolic syndrome.^( 28 )^

In the present study, the prevalence of increased NC was high (48.2%) and particularly more pronounced in men (60.4%) than in women (39.6%). A multicenter cross-sectional study of the Latin American Study of Nutrition and Health (ELANS) (n=2,370, 47.8% male) with participants from eight Latin American countries used the same cutoff points for increased NC (>39cm in men and >35cm in women). The prevalence of NC were high in Chile (56.9%), followed by Costa Rica (45.4%), Argentina (42.3%), Venezuela (42.0%), Peru (35.8%), Brazil (28.1%), Ecuador (29.9%), and Colombia (24.8%). The mean prevalence of increased NC was 37%, which was lower than that reported in the present study.^( 7 )^

A systematic review and meta-analysis on studies (85 studies, n=51,978)^( 9 )^ from the Latin America and the Caribbean estimated the mean NC and the prevalence of increased NC. The prevalence of increased NC ranged from 37% to 57.7% in the general population. However, the authors reported that the method to measure NC was not consistently reported, and there were several definitions of increased NC. Thus, although NC may be a new anthropometric indicator of different diseases, health outcomes, and lifestyles, NC has not been consistently studied, and the current evidence on NC in the region remains lacking.^( 9 )^

### Neck circumference and relative muscle strength

We found an association between low RMS and increased NC levels. A prevalence ratio of 1.26 and 1.73 times higher for high NC in the presence of low RMS, in men and in women, respectively, are associated with independent level of MVPA, demographic, lifestyle, and health conditions variables.

Handgrip strength is a well-established indicator of muscle strength and can be used to estimate sarcopenic obesity from adolescence.^( 5 )^ The authors concluded that owing to the demographic transition in several countries along with the aging of the population, obesity increases and an active lifestyle decreases. It is important to highlight that body adiposity is associated with low muscle strength. Both absolute HGS and RMS correlated with metabolic markers and obesity. However, the highest correlations were observed with RMS in both sexes after adjusting for age. This suggests that RMS is more adequate than absolute HGS for assessing the risk of chronic cardiometabolic diseases.^( 23 )^

Body mass, waist circumference, and NC correlated positively with absolute HGS, but inversely with RMS. Consistent with these findings and requiring careful interpretation, body size can influence HGS.^( 12 , 29 )^ Cross-sectional data from eight United Kingdom cohort studies (n=16,444 participants, 50-90 years) from Healthy Ageing across the Life Course, showed that body fat composition was associated with HGS in men only.^( 12 )^

A cross-sectional study conducted with 60 pre-menopausal women (mean age 33.9 years) to associate NC with RMS and cardiovascular risk factors in sedentary women found that women with increased NC had lower RMS values. The authors concluded that NC could be an important predictor of relative strength loss in sedentary, middle-aged women.^( 16 )^

The performance of hand muscle strength suggests a greater importance in preventing obesity. A study^( 30 )^ with a goal of verifying the association of RMS and obesity indicators in university professors identified an inverse correlation between RMS and waist circumference in both sexes.

The Korea National Health and Nutrition Examination Survey conducted on 2,451 participants (50+ years) found that low RMS was associated with higher cardiometabolic markers.^( 23 )^ Parameters related to obesity, such as insulin resistance and high-sensitivity C-reactive protein, were inversely and high-density lipoprotein cholesterol was positively associated with RMS in both sexes, even after adjusting for age and lifestyle factors. Relative muscle strength, a marker of metabolic syndrome, was analyzed using the Korea National Health and Nutrition Examination Survey.^( 31 )^ The study of 5,014 Korean adults aged ≥20 years (2,472 men and 2,542 women) showed a highly significant association between RMS and the risk of metabolic syndrome in Korean adults and may be a new biomarker for assessing the risk of diseases associated with obesity.^( 31 )^

For the Health, Well-Being, and Aging survey (SABE study)^( 13 )^ with a representative probabilistic sample of 1,046 residents (60+ years) from the city of São Paulo, Brazil, people with dinapenic abdominal obesity were more likely to experience fall and should be the target groups for the management of muscle weakness, falls, and the consequences of these events. With increasing age, there is a significant decrease in the level of PA^( 10 )^ which affects the reduction of strength and increase in muscle weakness, in addition to an increase in central body fat.^( 11 )^ This vicious cycle can lead to the muscle’s inability to respond to the required postural corrections because of the imbalance imposed by voluntary movements or external forces, subsequently increasing the risk of falls.^( 13 )^ This, in turn, may increase sedentary time even more longer because of the fear of falling. Physical activity programs, specifically resistance training, can improve frailty, sarcopenia, mobility limitations, central obesity, and other chronic conditions.^( 11 , 14 )^

In this context, PA and resistance training affect various variables of metabolic syndrome,^( 32 )^ central obesity, NC, and increases strength and muscle mass.^( 11 , 14 )^

### Neck circumference and moderate and vigorous physical activity

Our study revealed a significant association between MVPA and NC. After changes in RMS, demographic, lifestyle, and health condition variables, the association was significant in women (p<0.001) but not in men (p=0.627). This difference can be explained by the fact that men are more active than women^( 10 )^ and have higher NC.^( 7 , 33 )^

However, the results of the Longitudinal Study of Adult Health (ELSA-Brasil)^( 33 )^ after adjustments revealed that a one-centimeter increase in NC was associated with an increment of 3% and 5% in the risk of cardiovascular disease in 10 years (estimated by the Framingham Global Risk Score) in men and women, respectively. In the upper quartile of NC, men and women showed an increased risk of 18% and 35%, respectively. There was a greater association between the risk components (lifestyle and chronic diseases) and NC in women than in men; however, women have a lower median risk score of cardiovascular risk as manifested by higher HDL-cholesterol levels, lower mean systolic pressure, and lower prevalence of diabetes and smoking compared to that in men.^( 33 )^

The ELANS^( 7 )^ revealed a significant association between MVPA and NC in Costa Rica (OR= 0.980; 95%CI: 0.964-0.997, p=0.024) and Peru (OR= 0.989; 95%CI: 0.980-0.999, p=0.031). These findings were analyzed considering two hierarchical levels (country and region) and adjusted for sex, age, socioeconomic level, and educational level. However, no significant association was found between MVPA and NC in Argentina (p=0.490), Brazil (p=0.214), Chile (p=0.846), Colombia (p=0.105), Ecuador (p=0.643), or Venezuela (p=0.178). The authors found significant associations between MVPA and NC in adolescents and adults (15-65 years old) in eight countries in Latin America, with measured PA. They conclude that more research is needed to understand the associations and differences between countries.

With the goal of comparing PA and sedentary behavior associations with body composition in Latin American countries, the results of the ELANS study (n=2,368 participants, 51.9% women, aged 15-65 years)^( 15 )^ differed from our study. Moderate and vigorous physical activity were not significantly associated with NC. However, sedentary behavior was positively associated with NC even after adjusting, for confounding variable such as sex. Thus, sedentary behavior, different from non-active MVPA (<150minutes/week), may have an even greater impact on NC.

### Study limitations

This study has some limitations. First, the analysis is cross-sectional, and it is not possible to establish a cause-and-effect mechanism between the associations. Second, losses and refusals were more prevalent (p<0.05) among participants with low RMS, which could have underestimated the estimates reported in this study. However, this could further enhance the results obtained here. Third, the prevalence of MVPA may present some degree of bias because it was estimated in a self-reported manner through a questionnaire. However, this could further enhance the results obtained here. Despite these limitations, this study has some strengths, especially the number of participants and comparable data collection protocols. This study was conducted on a population-based sample representative of the city of Pomerode, Santa Catalina; and to the best of our knowledge, this is the first study that estimates the prevalence of NC associated with RMS and MVPA among individuals living in a community in Pomerania in Brazil.

## CONCLUSION

The present study showed that men and particularly women, with higher neck circumference values, had lower levels of moderate and vigorous physical activity and relative muscle strength. Thus, this study highlights the need to implement physical activity programs on muscle strength training to prevent and treat increased neck circumference and decreased relative muscle strength. We suggest using neck circumference in clinical health assessment as a measure of estimating excess fat in the upper body since it is easy to perform and has minimal need for material resources and equipment, in addition to lower patient exposure. Moreover, it serves as a factor for preventing low levels of physical activity and loss of relative muscle strength and can be applied to monitor active lifestyle and loss of functional physical capacity.
